# Time from Self-Detection of Symptoms to Seeking Definitive Care among Cervical Cancer Patients

**DOI:** 10.31557/APJCP.2020.21.11.3301

**Published:** 2020-11

**Authors:** Shivaraj Nallur Somanna, Srinivasa Nandagudi Murthy, Ramesh Cheluvarayaswamy, Nea Malila

**Affiliations:** 1 *Department of Community Medicine, Ramaiah Medical College, Bengaluru, India.*; 2 *Department of Epidemiology and Biostatistics, Kidwai Memorial Institute of Oncology, Bengaluru, India. *; 3 *Finnish Cancer Registry, Finland. *

**Keywords:** Cervical cancer, time interval, cancer symptoms, seeking cancer care

## Abstract

**Background:**

India had the burden of 97,000 new cases of cervical cancer with 60,000 deaths accounting nearly one-third of global cervical cancer deaths during the year 2018. Cervical cancer is the leading cause of cancer mortality in India. The present study aims to estimate the time interval between self-detection of cervical cancer symptoms and seeking care and different barriers for the possible time lag in seeking care.

**Methods:**

A cross-sectional study was undertaken from April 2017 to September 2017 in a regional cancer centre in the south of India. The centre has both a population and a hospital-based cancer registry. Cervical cancer cases (N= 210) with histological confirmation were interviewed at the hospital using a pre-tested semi-structured questionnaire.

**Results:**

The median time interval between the self-detection of cervical cancer symptoms and first contact with the general physician was 80 [IQR 45-150] days. The overall median time interval between the self-detection of symptoms to the initiation of primary treatment was 123[IQR 83-205] days. The major perceived reason for not seeking medical care was a lack of awareness in identifying cervical cancer symptoms in 183(92.9%) women.

**Conclusion:**

The median time of 80 days was observed from the self-detection of cervical cancer symptoms to the first contact with a general physician. Lack of awareness of patients pertaining to cancer symptoms was the major concern in seeking cancer care.

## Introduction

Globally, an estimated 570,000 new cases and 311,000 deaths has been reported due to cervical cancer in 2018 and it is the fourth most common cancer in women. The Incidence and mortality rank second behind breast cancer in lower human development index (HDI) settings. Eight out of 10 women diagnosed and nine of 10 women who die from cervical cancer live in a low- or middle-income countries (Bray et al., 2018) “ Cervical cancer continues to be a major public health problem affecting middle-aged women, particularly in less-resourced countries” (Arbyn et al., 2020). India had the burden of 97,000 new cases of cervical cancer with 60,000 deaths accounting nearly one third of global cervical cancer deaths during the year 2018 (“Globocan 2018,” 2018). Cervical cancer is the leading cause of cancer mortality in India which accounts for 17% of all cancer deaths among women aged 30 to 69 years.(Bobdey et al., 2016, “The Challenge Ahead,” 2014) One third of cervical cancer cases was reported among women aged 50 -59 years ( 27.4%) (Bobdey et al., 2016). The higher mortality and morbidity due to cancer is because most patients seek/get treatment at advanced stage and therefore have a higher risk of dying from the disease (Bray et al., 2018).

Cervical cancer is a preventable disease primarily because of its long lead time (women develop precancerous lesions years before it becomes cancerous). Cervical cancer can be detected in the pre-cancerous phase by screening and thereby the development of cancer prevented. Cervical cancer can also be treated successfully if diagnosed at an early stage. Routine screening by visual inspection of cervix through ‘See-and-treat’ approach among the female population has been suggested for early diagnosis and control of cervical cancer (Sankaranarayanan, 2012). Periodic Pap or HPV samples are difficult to organize in low- or middle-income countries like India and therefore large-scale routine screening would be difficult and not affordable (“HTA_CaCx Screening in India.pdf,” n.d.) Millions of cancer patients could be saved from premature death and disability, if they had timely access to early detection and treatment (“WHO,” n.d.). Studies have shown that it is possible to reduce the need of aggressive treatment of cancer is detected at an early stage. A long interval between start of disease symptoms to having a definite diagnosis is highly critical and associated with low survival among cervical cancer patients.(Chen et al., 2019) 

In India, the present existing public health care delivery system is a three-level system. The primary level is primary health centres (PHCs). In India, PHCs focuses on eight elements of primary health care outlined in the Alma-Ata declaration and provides mainly outpatient services. In the secondary level the district hospitals provide outpatient, inpatient and emergency services. At the tertiary level, medical college hospitals, apex institutions and regional cancer centres provide specialized services. Under the programme of “National programme for prevention and control of cancer, diabetes, cardiovascular diseases and stroke (NPCDCS)”, cancer screening is done at the primary level by visual inspection of the cervix with acetic acid (VIA) and those who are suspected for cancer are referred to secondary or tertiary care level for further management. In addition, a health programme for non-communicable diseases is carried out with a cancer care component implemented at all the health care levels (Gulia et al., 2016) 

This study aims to estimate the time interval between self-detection of cervical cancer symptoms and seeking for care and different barriers for the possible time lag in seeking care.

## Materials and Methods

**Figure 1:**
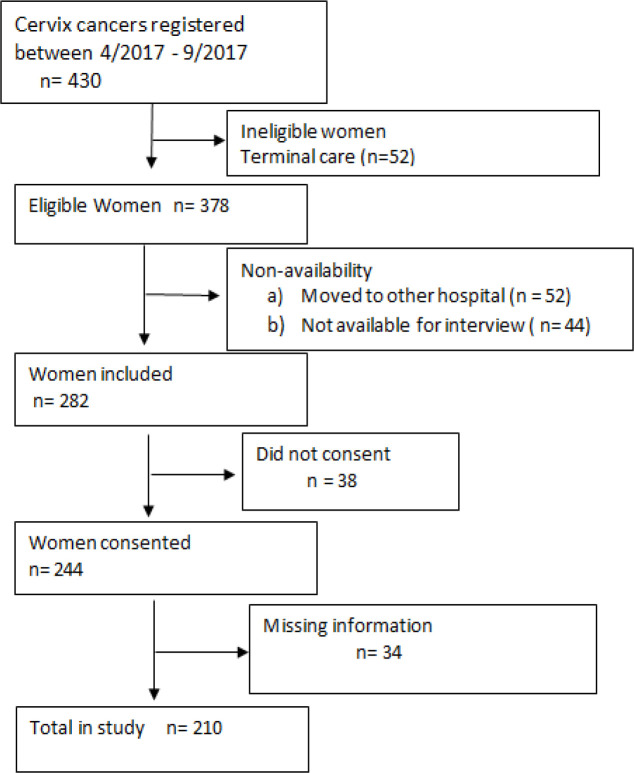
Recruitment of Participants for the Study

The data was collected from a regional cancer centre from south India, Bengaluru, which has a population-based cancer registry (PBCR) and a hospital-based cancer registry (HBCR). This tertiary care centre has state of art facilities for diagnosis, treatment and research. Cervical cancer cases with histological confirmation were recruited from the hospital-based cancer registry of this Institution during the time period of April 2017 to September 2017 using cross sectional study design. We identified 430 cervical cancer patients during the period from April 2017 to September 2017. Of these, 52 were ineligible due to referral to terminal/palliative care. Of 378 eligible women, 96 were not included for the analysis as patients moved to different hospital or not being available for interview, and 38 were not willing to participate in the study. In the 244 patients who gave their consent, 34 patient’s had incomplete information in the records. Finally, 210 patients were included for the present study (Figure 1) 

The cervical cancers were mostly squamous cell carcinomas (SCC) and few adenocarcinomas (AC). They were divided in two stage based on FIGO classification (Bhatla et al., 2019). Stage I and II were categorized as early stage; III and IV were considered as late stage of cervical cancer. 

Patient delay in seeking for medical care refers to a long interval between detecting of first/initial symptoms and seeking for appointment with a general physician. The patients were further divided into two groups, those who sought medical care within 90 days and those who sought care 90 days and above (patient delay) after self-detection of the first symptoms of cervical cancer. 

Sample size was calculated based on the study conducted by Rudd et al., (2017) where 39.3% patients had delayed diagnosis from the onset of self-detection of cervical cancer symptoms. In this study the sample size was estimated for patient related delay, from symptoms to first contact with a general practitioner, as it was our major concern. To get an absolute precision of 6.6% with 95% confidence level, the study required a minimum sample of 210 subjects. Based on hospital record collection, it was noted that six months’ time period was sufficient to achieve the required sample size. 

Expert opinion from oncologists, biostatisticians and epidemiologists were obtained using the Delphi technique (Skulmoski et al., 2007). After obtaining the written informed consent, patients were interviewed in person using the questionnaire and their medical records was used to collect the baseline information as well as key components of the time interval between self-detection of cervical cancer symptom to seeking medical care. More details of the collection of data and the different components of time intervals are described elsewhere.(Somanna et al., 2020)

Independent variables included socio-demographic characteristics, such as age at diagnosis, availability of health insurance, place of residence (rural/urban), history of cancer literacy, and financially dependent for livelihood and clinical variables, such as personal history of cancer, and family history of cancer.

The time interval between various stages was measured in days as reported by the patients and by review of hospital case sheets as indicated in earlier study.(Somanna et al., 2018) 

a) Time interval between self-detection of symptoms and the first presentation at general practitioner (T1). 

b) Time interval from referral by the general practitioner to attending tertiary care centre (T2). 

c) Time interval from first consultation at tertiary care for diagnosis to definite diagnosis date (T3). 

d) Time interval from definite diagnosis to initiation of primary treatment (T4). 

e) Total time elapsed from self-detection of symptoms to initiation of primary treatment (T5).

*Statistical methods*

Descriptive statistics of time intervals were summarized with median and inter-quartile range as data were not normally distributed (Kolmogrov-Smirnov and Shapiro test). Univariate odds ratios (OR) with 95% confidence intervals (CI) were estimated between the factors and patient delay. The patient delay was categorised into < 90 days and ≥ 90 days. Considering ≥ 90 days as patient delay, the association of various factors were estimated considering the low risk group as the reference. Variables showing a univariate association with patient delay (at P < 0.20) were included in multivariate unconditional logistic stepwise forward regression model to adjust for confounders. The analysis was carried out using SPSS Inc. Released 2009. PASW Statistics for Windows, Version 18.0. Chicago. 

Ethical approval was sought from the ethical review committee of the study Institution. 

## Results

The distribution of socio-demographic and clinical data of the 210 cervical cancer study subjects are presented along with data pertaining to the cases not enrolled for analysis (Table 1). Among 210 enrolled patients, the majority 106 (50.5%) of women were between the age 46 to 59 years where in not enrolled patients 89 (40.4%) were of same age group. It was noted 167 (63.0%) and 179 (81.4%) were not literate among enrolled and respectively. Among enrolled patients, 150 (71.4%) were from rural areas and 158 (71.4%) were financially dependent for livelihood on the family. Only 19 women (9.0%) had any health insurance policy. The majority of patients 197 (93.8%) were not aware of symptoms of cervical cancer and only 7 (3.3%) patients had a family history of cancer.

Of the symptoms which led for seeking medical care, the majority of women, 172 (81.9%), indicated lower abdominal pain, and 137 (65.2%) of the respondents experienced abnormal vaginal discharge/bleeding. Among all patients, 106 (50.5%) were diagnosed at an early stage of the disease (stages I, IIA and IIB).

The median time interval between self-detection of cervical cancer symptoms and first contact with general physician (T1) was 80 [IQR 45-150] days, while 74 patients (35.2%) had a time lag of 90 days or more. The median time taken for reaching diagnosis after the first contact (T2) was 25 [IQR 15 - 45] days. The overall median time between self-detection of symptoms to initiation of primary treatment (T5) was 123[IQR 83-205] days. The majority of patients, 145 (66.2%), had initiation of primary treatment only after 90 days from self-detection of cervical cancer symptoms (Table 2). 

Factors associated with delay in seeking medical care of 90 days and more (patient delay) were analyzed in relation to socio-demographic characteristics and clinical symptoms (Table 3). In multivariate analysis after adjusting to all the variables in the study the odds of patient delay in the age group ≥ 60 years was 3.2 times higher compared to patients in the age group below 45 years (95% CI: 1.2 – 8.4). The other factors which were associated with patient delay were being not literate (OR 2.3, 95% CI: 1.0 – 5.4), from rural area (OR = 2.3, 95% CI: 1.1 – 4.9), being financially dependent for livelihood (OR = 4.5, 95% CI: 1.9 – 10.6) and not having any abnormal vaginal discharge / bleeding (OR = 2.4, 95% CI: 1.3 – 4.6). Family history of cancer, presence of any health insurance, knowledge regarding cancer cure by early seeking for care, knowledge of symptoms of cervical cancer and having lower abdominal pain was not statistically related to delay in seeking medical care. 

Information was sought regarding perceived reasons from 197 (93.8%) patients who did not seek care within one week from self-detection of cancer symptoms. The major reasons (table 4) were lack of awareness in identifying cervical cancer symptoms in 183 (92.9%) women and 120 (60.9%) patients assuming that the symptom would resolve by itself. The other common reasons were absence of pain, changes in the body attributed to common illness and vague symptoms. 

The most common reason for delay in seeking for definite diagnosis at tertiary care hospitals was due to non-affordability 82 (41.6%). The other common reasons were visiting multiple medical practitioners before presenting at tertiary care 60 (30.1%) and not suspected for cancer by physician at first contact. 

The delay in seeking for treatment after diagnosis at tertiary care was due to long treatment procedure 57 (27.1%), fear of treatment 50 (23.8%), financial dependence on the family 48 (22.9%), fear of disfigurement of the body 26 (12.4%) and stigma attached with the disease (Table 4).

**Table 1:** Distribution of Socio-Demographic, Clinical and Other Variables Among the Subject Enrolled and Not Enrolled to the Study

Variable	Subjects enrolled n (%)	Subjects not enrolled n (%)
Age (in years)		
≤ 45	61 (29.0)	45 (20.5)
46 – 59	106 (50.5)	89 (40.4)
≥60	43 (20.5)	86 (39.1)
Literacy status		
Not literate	167 (63.0)	179 (81.4)
Literate	43 (37.0)	41 (18.6)
Area of Residence		
Rural	150 (71.4)	169 (76.8)
Urban	60 (28.6)	51 (25.2)
Financial Dependence for livelihood		
Yes	158 (75.2)	-
No	52 (24.8)	-
Family history of Cervical cancer		
Yes	7 (3.3)	-
No	203 (96.7)	-
Any Health Insurance		
Yes	19 (9.0)	-
No	191 (91.0)	-
Knowledge regarding cancer cure by early seeking for care		
Yes	9 (4.3)	-
No/Don’t Know	201 (95.7)	-
Family History of cervical cancer		
Yes	7 (3.3)	
No	203 (96.7)	
Awareness of symptom of Cervical Cancer		
Yes	13 (6.2)	-
No	197 (93.8)	
Abnormal vaginal discharge / Bleeding		
Yes	102 (48.6)	
No	108 (51.4)	
Lower abdominal pain		
Yes	38 (18.1)	
No	172 (71.9)	
Clinical Staging of the disease		
Early stage (IIA + IIB)	106 (50.5)	-
Late stage (IIIA+ + IV)	104 (49.5)	-

**Table 2:** Distribution of Time Interval Between Self-Detection of Cancer Symptoms to Various Stages (T1 To T5) for Seeking Treatment

Time interval between	Median [IQR 25-75] (in days)
Self-detection of symptoms to first presentation for medical care (T1)	80 [45, 150]
First contact to presentation at tertiary care (T2)	25 [15, 45]
Initial Presentation at tertiary care to definite diagnosis (T3)	11 [7, 18]
Definite diagnosis to Initiation of primary treatment (T4)	14 [10, 24]
Total time from onset of symptom to initiation of primary treatment (T5)	123 [83, 205]

**Table 3:** Factors Associated with Delay in Seeking Medical Care: Univariate and Multivariate Logistic Regression Results

Characteristic	Time in seeking medical care (T1) Patient delay			Univariate Odds ratio (95%CI)	Adjusted Odds ratio (95%CI)
	≥ 90 days n= 74	< 90 days n= 136	Total n = 210		
Age (in years)					
≥ 60	19 (25.7)	24 (17.6)	43 (20.5)	2.7 (1.1, 6.2) *	3.2 (1.2, 8.4) *
46 – 59	41 (55.4)	65 (47.8)	106 (50.5)	2.1 (1.0, 4.3)	1.3 (0.6, 2.9)
≤ 45	14 (18.9)	47 (34.6)	61 (29.0)	1	1
Literacy Status					
Not literate	65 (87.8)	102 (75.0)	167 (63.0)	2.4 (1.1, 5.3) *	2.3 (1.0, 5.4) *
Literate	9 (12.22)	34 (25.0)	43 (37.0)	1	1
Area of residence					
Rural	61 (82.4)	93 (68.4)	150 (71.4)	2.1 (1.1, 4.4) *	2.3 (1.1, 4.9) *
Urban	13 (17.6)	43 (31.6)	60 (28.6)	1	1
Financial dependence for livelihood					
Yes	65 (87.8)	93 (68.4)	158 (75.2)	3.3 (1.5, 7.3) *	4.5 (1.9, 10.6) *
No	9 (12.2)	43 (31.6)	52 (24.8)	1	1
Family history of Cancer					
Yes	2 (2.7)	5 (3.7)	7 (3.3)	0.7 (0.1, 3.9)	0.5 (0.1, 2.8)
No	72 (97.3)	131 (96.3)	203 (96.7)	1	1
Any health Insurance					
No	70 (94.6)	121 (89.0)	191 (91.0)	2.2 (0.7, 6.8)	3.2 (0.9, 11.3)
Yes	4 (5.4)	15 (11.0)	19 (9.0)		1
Knowledge regarding cancer cure by early seeking for care					
No	69 (93.2)	130 (95.6)	201 (95.7)	0.6 (0.2, 2.2)	0.9 (0.8, 1.1)
Yes	5 (6.8)	6 (4.4)	9 (4.3)	1	1
Awareness of symptom of Cervical Cancer					
No/Don’t Know	70 (94.6)	127 (93.4)	13 (6.2)	1.2 (0.4, 4.2)	0.6 (0.2, 2.5)
Yes	4 (5.4)	9 (6.6)	197 (93.8)	1	
Abnormal vaginal Discharge / Bleeding					
No	47 (63.5)	61 (44.9)	108 (51.4)	2.1 (1.2, 3.8) *	2.4 (1.3, 4.6) *
Yes	27 (36.5)	75 (55.1)	102 (48.6)	1	1
Lower abdominal pain					
No	63 (85.1)	109 (80.1)	172 (81.9)	1.4 (0.7, 3.1)	0.9 (0.4, 2.2)
Yes	11 (14.9)	27 (19.9)	38 (18.1)	1	1

**Table 4:** Distribution of Perceived Reasons for Delay in Seeking Medical Care at Various Stages (T_1_ – T_4_)

Delay Stage	Perceived Reasons	Frequency (%)
Self-detection of symptoms to first contact (N = 197) *	Lack of awareness of cervical cancer symptoms	183 (92.9)
	Belief that cancer symptom would resolve by itself	120 (60.9)
	Absence of pain	60(30.5)
	Changes in the body attributed to common illness	63 (32.0)
	Vague symptoms	49 (24.9)
	Shame associated with disease	51 (25.9)
	Fear of surgery	13 (6.6)
	Financial problems	59 (28.1)
Presenting at tertiary care for Diagnosis after first contact (N= 197) *	Visited multiple medical practitioners before presenting at tertiary care (primary care providers)	60 (30.1)
	Affordability	82 (41.6)
	Not suspected for cancer at first contact	41 (20.8)
	Nobody to accompany	36 (17.1)
Treatment after diagnosis (N = 132) #	Fear of treatment	50(23.8)
	Dependent on the family	48(22.9)
	Treatment would disfigure the body	26(12.4)
	Affordability	47(22.4)
	Long treatment procedure	57(27.1)
	Treatment Not comfortable	30(14.3)
	Stigma	32(15.2)
	Distance of Hospital	15(7.1)
	Loss of wage	29(13.8)

*, Responses were elicited from those who had not sought specific care for more than one week from the onset of symptoms; #, Responses was elicited from those who had not sought treatment on specified time by doctor

## Discussion

The study centre was a regional cancer care centre having both population and hospital-based cancer registry and where a majority of cervix cancer patients from the entire state seek cancer care. The median age in enrolled and not enrolled patients was 50 and 56 years, respectively. Among all the patients, the proportion of not literate patients was higher (81%) in not enrolled patients compared to enrolled patients (63%). The study by Deshmukh and Rathod, (2017) observed that the average age of the patients was 57 years and 81% were not literate. Our result concur with the study by Panda et al., 2019 where the average age of the study participants was 52.1 ± 10.9 years. 

The overall median time between the self-detection of cervical cancer symptoms and initiation of primary treatment was 123[IQR 83-205] days and the median time taken for first contact with general physician from self-detection of symptoms was 80 [IQR 45-150] days. Overall, 66% women delayed (≥ 90 days) for initiation of primary treatment from the onset of symptoms which is potentially concerning, because longer duration between diagnosis and treatment is related with higher risk of death. Among patients who started their treatment after an interval of >180 days, the risk of death was reported to be 36% higher compared to those who started treatment within 6 months from diagnosis (Chen et al., 2019) 

The present study assessed the time interval between self-detection of symptoms to treatment of cervical cancer and patient delay. The median time interval between self-detection of symptoms and the first presentation at general practitioner (T1) was 80 days with 35.4% of the patients reporting 90 days or more. The present study observation was consistent with a study conducted in India on patients attending tertiary care centre, showing that 39% of patients had a diagnostic delay (Panda et al., 2019) The median time between symptoms and presenting at health care provider was 69 days from two tertiary care centres in Nepal (Gyenwali et al., 2014). It was noted from the study by Lim et al., (2014) that only 28% of patients had a delay of more than 3 months in all national health service (NHS) hospitals diagnosing cervical cancer in England. There was a mean delay of 23.1 weeks between onset of symptoms from the study in tertiary care centre of Sothern Malawi (Rudd et al., 2017). Results from our study were in line with studies from low- or middle-income countries but differed from studies in developed countries. This difference may be partially due to regular screening programs in many developed countries and differing rates of literacy (IARC, 2005). 

Overall median time from self-detection of symptoms to initiation of primary treatment (T5) was 123 days in the current study. Another study by Henriques, (2016) in India showed on an average six months duration from first symptom to cervical cancer treatment. Such a long time may be explained by the fact that lack of awareness of patients pertaining to cancer symptoms was high (93%) in present study. In another study, women who delayed, had mistaken the symptoms of cervix cancer as symptoms of sexually transmitted diseases (STD) and were scared to report (Deshmukh and Rathod, 2017). This shows that the lack of awareness of cervix cancer symptoms is a major factor and warrants improvement in public awareness. Our study results concur closely with other studies from similar settings and in low- or middle-income countries (Benemariya et al., 2018; Gyenwali et al., 2014).

The patients who were aged 60 years and above, residing in rural areas, not literate, financially dependent for livelihood and did not had abnormal vaginal discharge/ bleeding were independently associated with patient delay. Similar results were observed in a study from Nepal where not literate was found to be independently associated with late diagnosis and women having abnormal vaginal bleeding as symptom was protective factor for late diagnosis (Gyenwali et al., 2013). Previous studies from low- or middle-income countries also concur with our result (Dunyo et al., 2018)(Ouasmani et al., 2016)(Henriques, 2016).

The present study has been conducted in a population-based cancer registry (PBCR) where the data are included in International Agency for Research on Cancer (IARC) and data collection procedure involves standard quality control measures. The study included only patients who had been diagnosed with standard clinical and histopathological criteria. The information was obtained using pre-tested validated questionnaire by the investigator. Additional information pertaining to clinical evaluation and histological diagnosis were obtained from case sheets. All efforts were made to reduce the recall bias by local events calendar and standardization of terms employed in the questionnaire. 

*Limitations*

Possible selection bias could have occurred as the subjects who were not enrolled for the study due to various reasons were older women and not literate compared to subjects in the study. Socio-demographic profile of population who availed the services at this centre may be different from private tertiary cancer care centre which would biases the result. The sample in study represent only those who availed the treatment at the centre and does not represent the entire population of cervical cancer patients. Thus, it may have some restrictions for external generalization. Possibility of recall bias exist as past history of cancer symptoms were assessed retrospectively. 

In conclusion, the median time of 80 days was observed from self-detection of cervical cancer symptoms to first contact with general physician. Further, a median time of another 45 days was observed for obtaining primary treatment. Lack of awareness of patients pertaining to cancer symptoms was the major concern in seeking cancer care. Interventions like raising awareness of cervical cancer symptoms and improving health seeking behavior of women should be focused.
